# Clinical Outcomes of Transdiscal Screws for Thoracolumbar Spinal Fractures with Marked Anterior Distraction Gap Accompanied by Diffuse Idiopathic Skeletal Hyperostosis

**DOI:** 10.3390/medicina61101874

**Published:** 2025-10-19

**Authors:** Ryo Ugawa, Yoshihiro Fujiwara, Toshiyuki Matsumoto

**Affiliations:** Department of Orthopaedic Surgery, Kochi Health Sciences Center, 2125-1 Ike, Kochi City 781-8555, Japan

**Keywords:** diffuse idiopathic skeletal hyperostosis (DISH), spinal fractures, thoracolumbar spine, transdiscal screw for diffuse idiopathic skeletal hyperostosis (TSD), posterior fixation, anterior fracture gap

## Abstract

*Background and Objectives*: Diffuse idiopathic skeletal hyperostosis (DISH)-related spinal fractures with marked anterior distraction are highly unstable and pose substantial surgical challenges. The transdiscal screw for diffuse idiopathic skeletal hyperostosis (TSD) technique has been proposed to enhance fixation strength by penetrating adjacent vertebral endplates; however, its clinical utility in large-displacement cases remained unclear. *Materials and Methods*: In this retrospective study, we reviewed 21 patients with thoracolumbar DISH-related fractures and an anterior fracture gap ≥ 15 mm, who underwent posterior fixation between 2010 and 2024. 11 patients underwent TSD fixation (TSD group), and 10 underwent conventional fixation without bilateral TSD (control group). *Results*: The mean number of fused segments did not differ significantly between the groups (5.0 ± 1.4 vs. 5.0 ± 1.3, *p* = 0.43). Operative time was comparable (164 ± 57 vs. 168 ± 60 min, *p* = 0.90). Blood loss tended to be lower in the TSD group (306 ± 334 vs. 528 ± 658 mL, *p* = 0.33). For fracture-gap reduction, the TSD group improved from 17.4 ± 2.3 mm preoperatively to 13.8 ± 4.4 mm postoperatively and 2.0 ± 3.6 mm at final follow-up, while the control group showed less reduction (16.8 ± 2.2, 15.4 ± 1.4, and 7.0 ± 9.1 mm, respectively). Screw loosening occurred in three TSD patients and six controls (*p* = 0.13). All patients in the TSD group achieved bone union without reoperation, whereas four controls experienced implant backout, three required reoperation, and two failed to achieve bone union (*p* = 0.035). *Conclusions*: Posterior fixation using TSD provided reliable stability, maintained reduction, and reduced the risk of implant failure compared with conventional fixation in highly unstable DISH-related fractures with anterior distraction. Although larger prospective studies are needed, TSD may represent a valuable surgical option for this challenging patient population.

## 1. Introduction

Diffuse idiopathic skeletal hyperostosis (DISH) is a common disorder in the elderly, characterized by extensive ossification of the spinal ligaments and ankylosis across multiple motion segments. Spinal fractures in patients with DISH are often highly unstable, even after low-energy trauma, because rigid ankylosed segments create long lever arms. These injuries are associated with a substantial risk of neurological deterioration and poor clinical outcomes [1,2].

Fractures with a marked anterior distraction gap pose a greater challenge. The combination of poor bone quality, excessive instability, and large fracture gaps often compromises the effectiveness of conventional fixation, increasing the risk of implant loosening or nonunion. Thus, achieving secure and durable stabilization in this setting remains a critical clinical issue.

Transdiscal screw for diffuse idiopathic skeletal hyperostosis (TSD) fixation has been proposed as a strategy to enhance stability in DISH by penetrating the adjacent vertebral endplates, even in osteoporotic spines of elderly patients [3]. This approach may be particularly advantageous in cases of DISH-related fractures with severe anterior distraction, where stronger purchase and construct stability are required.

The present study reports the clinical outcomes of TSD fixation for unstable DISH-related spinal fractures with a marked anterior distraction gap and evaluates its utility as a surgical option in this challenging patient population.

## 2. Materials and Methods

**Figure 1:**
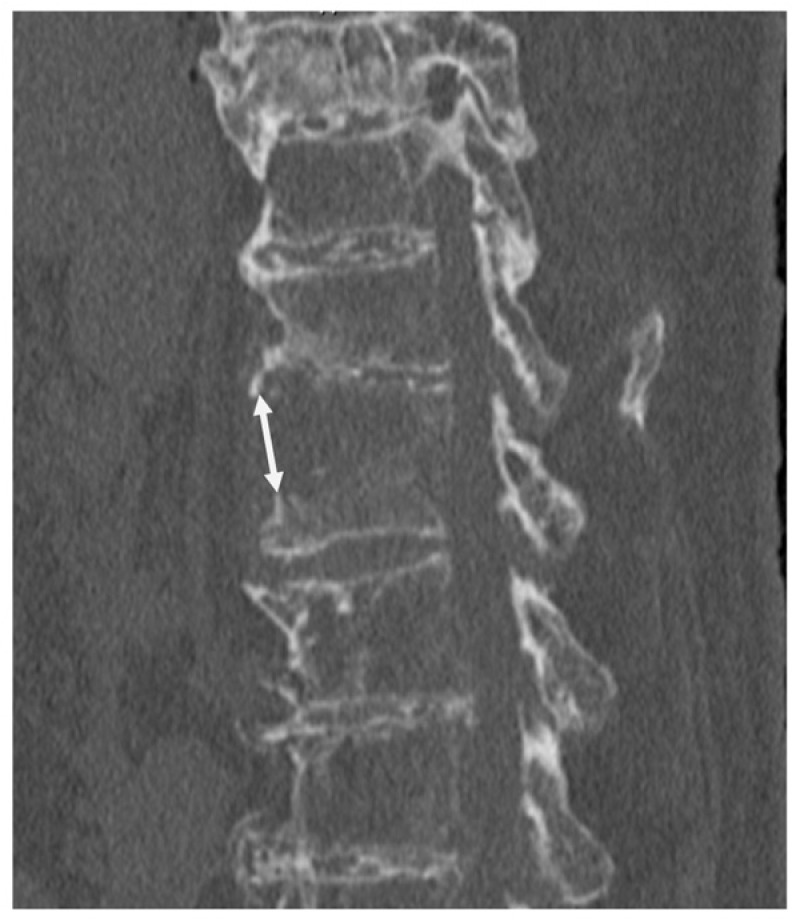
A two-headed arrow indicates an anterior fracture gap on preoperative imaging.

**Figure 2:**
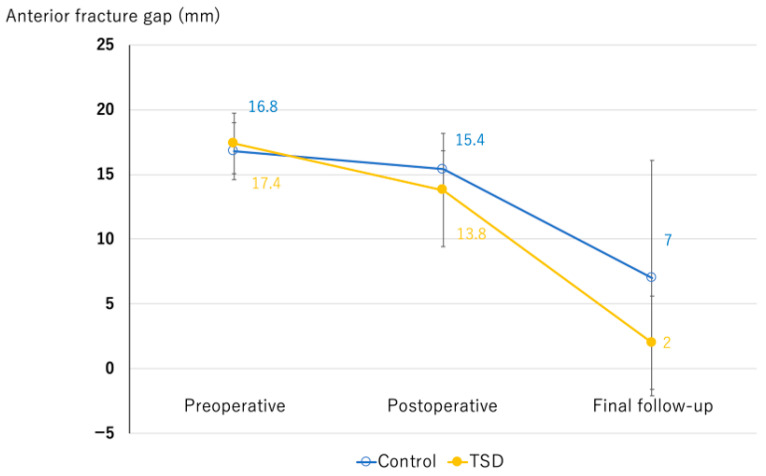
The mean anterior fracture gap in the TSD group and control group at preoperatively, postoperatively, and final follow-up. *p* values at each time point were 0.58, 0.29, and 0.11.

This study was approved by the institutional ethics committee (No. 251035). Informed consent was obtained from all the patients involved in the study. A total of 143 patients admitted to our hospital for the treatment of thoracolumbar spine fractures accompanied by DISH between December 2010 and February 2024 were retrospectively assessed. Among them, 21 patients met the following inclusion criteria: (1) an anterior fracture gap of ≥15 mm or on preoperative imaging (Figure 1), (2) treatment with posterior fixation alone without anterior reconstruction, and (3) postoperative follow-up of at least 8 months. These 21 patients were further divided into two groups according to the use of TSD. The TSD group comprised 11 patients who received penetrating endplate screws both cranially and caudally to the fracture level. The control group included 10 patients who either did not receive penetrating endplate screws or received them on only one side (cranial or caudal). In the early period of this study, conventional posterior fixation without TSD was performed; however, since 2020, TSD fixation has been routinely applied for DISH-related thoracolumbar fractures based on its biomechanical advantages. Comparative analyses were performed between the two groups (Table 1). Outcome measures included the number of fused segments, operative time, intraoperative blood loss, postoperative changes in the anterior fracture gap, degree of neurological recovery, presence of screw loosening, achievement of bone union, and requirement for additional surgery.

### 2.1. Operation Procedure

The entry point for the TSD was just below the pedicle (toward the 5 o’clock position for the right pedicle and toward the 7 o’clock position for the left pedicle). Regarding the TSD trajectory, the screw passes through the pedicle in the cranial direction to penetrate two vertebral endplates: the cranial vertebral endplate of the vertebrae through which the screw enters and the caudal endplate of the adjacent cranial vertebra [3]. Percutaneous pedicle screws (PPS) were used for spinal implants to minimize surgical invasiveness in all patients. A navigation system guided screw insertion. Intraoperative images were obtained using a C-arm scanner (Cios Spin; Siemens, Munich, Germany), and intraoperative CT images were obtained using a navigation system (Kick2; BrainLab, Munich, Germany). Various commercially available percutaneous pedicle screw systems were used depending on the surgical period and implant availability, including Voyager (Medtronic, Memphis, TN, USA) and Ennovate (B. Braun Aesculap, Tuttlingen, Germany).

### 2.2. Statistical Analysis

Continuous variables are expressed as mean ± standard deviation, and categorical variables are expressed as absolute numbers or percentages. Differences between the TSD and non-TSD groups were analyzed using Student’s *t*-test or the Mann–Whitney U test for continuous variables and the chi-square test or Fisher’s exact test for categorical variables, as appropriate. Statistical significance was set at *p* < 0.05. All statistical analyses were performed using EZR software version 1.68 (Saitama Medical Center, Jichi Medical University, Saitama, Japan).

## 3. Results

The mean number of fused segments in the TSD and control groups did not differ significantly (5 ± 1.4 vs. 5 ± 1.3 segments, *p* = 0.43). The mean operative time was 164 ± 57 min in the TSD group and 168 ± 60 min in the control group (*p* = 0.90). The mean intraoperative blood loss was 306 ± 334 mL in the TSD group and 528 ± 658 mL in the control group (*p* = 0.33).

**Figure 3:**
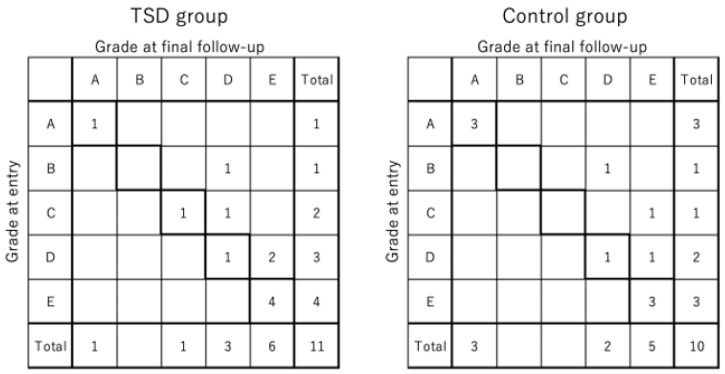
The distribution of Frankel grades at entry and final follow-up.

The mean anterior fracture gap in the TSD group was 17.4 ± 2.3 mm preoperatively, 13.8 ± 4.4 mm postoperatively, and 2.0 ± 3.6 mm at the final follow-up. The corresponding values in the control group were 16.8 ± 2.2 mm, 15.4 ± 1.4 mm, and 7.0 ± 9.1 mm, respectively. No significant differences were observed between groups at any time point (*p* = 0.58, 0.29, and 0.11) (Figure 2). The distribution of Frankel grades at baseline and final follow-up is shown in Figure 3. At the final follow-up, screw loosening was observed in three patients in the TSD group and in six patients in the control group, with no statistically significant differences (*p* = 0.13).

All patients in the TSD group achieved bone union, and no reoperations were required. In contrast, implant back-out occurred in four patients in the control group (*p* = 0.035), three of whom required reoperation; additionally, two patients in the control group failed to achieve bone union at the final follow-up (Table 2).

### 3.1. Case Presentation 1

**Figure 4:**
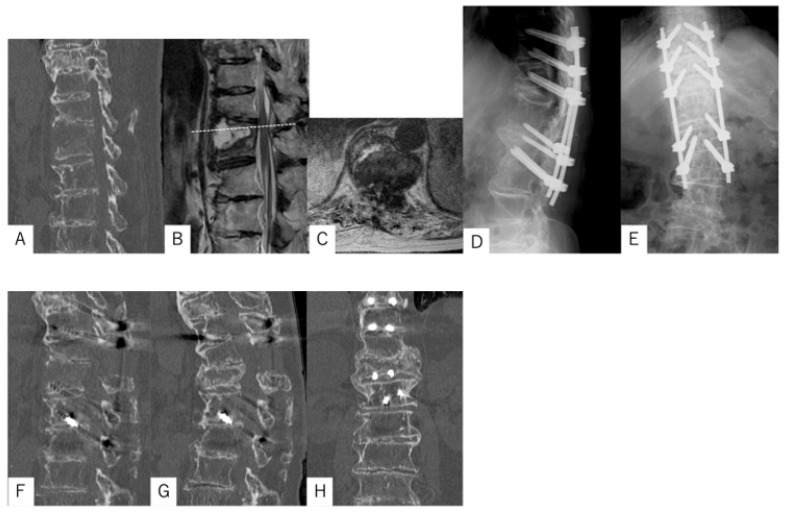
Spinal fracture case with DISH involving an 89-year-old woman. (**A**) Initial CT image showing a marked anterior distraction gap (19 mm) at Th12. (**B**) Initial sagittal magnetic resonance imaging (MRI) image. (**C**) Initial axial MRI at the level indicated by the dotted line in panel (**B**), showing spinal canal stenosis at the Th11/12 level. (**D**) Postoperative posteroanterior radiogram. (**E**) Postoperative lateral radiogram. Posterior fixation was performed from Th9 to L2 (five levels) using TSDs, with a laminectomy from Th11 to Th12. (**F**) CT image 1 week postoperatively showing a residual anterior gap of 14 mm. (**G**) Sagittal CT 12 months postoperatively showing complete closure of the gap (0 mm). (**H**) Coronal CT 12 months postoperatively demonstrating sufficient bone bridging and union with no implant loosening.

The patient was an 89-year-old woman who fell, sustained injuries, and was transported to our hospital. On initial examination, she reported severe back pain, numbness in the genitals and legs, and difficulty in urinating. Muscle strength was decreased by manual muscle testing (MMT) grade 4 out of 5 in the bilateral quadriceps and anterior tibialis muscles. A spinal fracture with a marked anterior distraction gap (18 mm) was found at Th12 on computed tomography (CT). The thoracolumbar spine, including the injury level, had extensive DISH, both cranially and caudally, and the amount of trabecular bone in the anterior-to-posterior column around the fracture site was significantly reduced. Magnetic resonance imaging (MRI) revealed spinal canal stenosis at the Th11/12 level. Posterior fixation from Th9 to L2 (five levels) using TSDs, along with laminectomy from Th11 to Th12, was performed in the prone position. Preoperatively, the 4-point spine frame was adjusted to prevent the fracture gap from expansion and optimize patient positioning. CT performed 1 week postoperatively showed a residual anterior gap of 14 mm. At 12 months post-surgery, the gap had disappeared (0 mm), with sufficient bone bridging and union achieved, and no implant loosening observed. The patient was able to walk with a cane and reported no back pain (Figure 4).

### 3.2. Case Presentation 2

**Figure 5:**
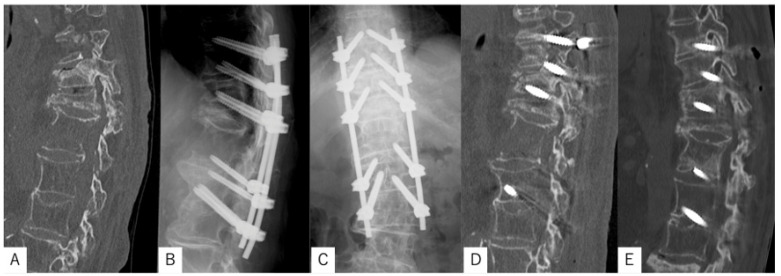
Spinal fracture case with DISH involving an 89-year-old woman. (**A**) Preoperative CT showing a marked anterior distraction gap (17 mm) at L1. (**B**) Postoperative posteroanterior radiogram. (**C**) Postoperative lateral radiogram. Posterior fixation was performed from Th10 to L3 (five levels) using TSDs. (**D**) CT 1 week postoperatively showing a residual anterior gap of 17 mm. (**E**) CT 9 months postoperatively showing complete closure of the gap (0 mm), with sufficient bone bridging and union and no implant loosening.

The patient was an 89-year-old woman who fell, sustained injuries, and was transported to our hospital. Upon initial examination, she reported severe back pain, with no evident neurological deficits. A spinal fracture with a marked anterior distraction gap (17 mm) was identified at L1 on CT. The thoracolumbar spine, including the injury level, had DISH cranially and caudally, with markedly reduced trabecular bone in the anterior–posterior column around the fracture site. Posterior fixation from Th10 to L3 (five levels) using TSDs was performed. Preoperatively, the 4-point spine frame was adjusted to prevent the fracture gap from expansion and optimize patient positioning. CT performed 1 week postoperatively showed a residual anterior gap of 17 mm. At nine months post-surgery, the gap had disappeared (0 mm), with sufficient bone bridging and union achieved, and no implant loosening observed. She had no back pain and no difficulty with activities of daily living (Figure 5).

## 4. Discussion

In the present study, we evaluated the clinical outcomes of TSD for unstable spinal fractures associated with DISH with marked anterior distraction. The key finding of this study was that all patients in the TSD group achieved bone union without requiring reoperation, whereas several patients in the control group developed implant backout, nonunion, or required reoperation. Although statistical significance was not observed for operative time, blood loss, fusion length, changes in the anterior gap, and frequency of screw loosening, likely due to the small cohort size, these results suggest that the TSD technique may confer biomechanical and clinical advantages in this challenging patient population.

Spinal fractures in patients with DISH are increasingly recognized as highly unstable injuries with a poor natural history [4]. The ankylosed spine behaves like a long lever arm, making even low-energy trauma capable of producing three-column injuries that resemble long-bone fractures [5]. Previous studies have reported high rates of fixation failure and pseudarthrosis following conventional posterior instrumentation, particularly when fracture distraction is pronounced [6]. Therefore, various techniques have been proposed to augment the fixation strength, including longer-segment fixation [7], cement augmentation [8,9], and combined anterior reconstruction [10]. However, these approaches may be associated with longer operative time, greater invasiveness, or increased morbidity in elderly patients with multiple comorbidities.

Several studies have reported that the use of PPS in DISH-related fractures significantly reduces surgical invasiveness compared with conventional open surgery, and PPS has therefore become widely accepted for managing these fractures [11,12]. TSD is based on PPS fixation. The trans-endplate pedicle screw insertion technique has been described as an alternative strategy to increase fixation strength by engaging cortical bone beyond the vertebral endplate [3,13]. Biomechanical studies demonstrated that this technique provided greater intraoperative screw insertion torque and pullout strength than conventional pedicle screws in ankylosed spines. According to biomechanical studies of vertebral endplates, the strength of the endplate increases toward the lateral portion of the thoracic spine, with similar results in the lumbar spine [14,15]. Because the trajectory of the TSD passes through the pedicle and traverses the lateral part of the vertebral endplate, it engages this stronger region and can therefore be considered an ideal screw trajectory. Furthermore, biomechanical studies on pedicle-screw length have shown that longer screws are associated with increased pullout strength and vertebral fixation stability [16]. From this perspective, insertion of the longest possible screw is considered important for enhancing screw fixation strength. The TSD trajectory inevitably extends into the adjacent vertebral body, resulting in a longer distance from the screw entry point to the anterior margin of the adjacent vertebral body compared with the conventional pedicle screw trajectory. Ikuma et al. reported that TSD allowed the insertion of longer screws than the conventional technique [3]. Our findings support these reports, showing that TSD fixation is associated with reduced screw loosening and more reliable bone union. Notably, no patient in the TSD group required reoperation, in contrast to the control group, in which implant failure and nonunion were common. These results suggest that TSD may offer particular benefits in cases with a severe anterior fracture gap, where conventional fixation may fail to provide sufficient stability. Several recent studies suggest that DISH is associated with inherently elevated osteogenic potential. For example, bone marrow mesenchymal stem cells (BMSCs) from DISH patients demonstrate stronger differentiation into osteoblasts compared to controls, with Galectin-3 and Wnt/β-catenin signaling implicated in this enhanced osteogenesis [17]. Genetic-epidemiological data also indicate that DISH is associated with increased bone mineral density and bone content across the skeleton, with genes related to bone formation (e.g., RUNX2, IL11) implicated in its pathogenesis [18]. In addition, histological observations in DISH patients reveal elevated expression of BMP-2, TGF-β, and factors associated with matrix calcification and endochondral ossification in heterotopic ossification sites [19]. Even when a residual fracture gap persisted immediately after fixation, the robust stability afforded by TSD, together with the hypertrophic osteogenic capacity associated with DISH, may have facilitated the favorable bone union observed in this series. This hypothesis is based on previous basic-science findings and that further histological and experimental studies are needed to validate the association between TSD fixation and enhanced osteogenesis.

However, this study has some limitations. First, it was retrospective in nature, and the small sample size limited statistical power, which may explain why differences between groups did not reach significance. Second, the minimum follow-up period was eight months, which, although sufficient to evaluate bone union, may be inadequate for assessing long-term complications, such as adjacent-segment degeneration or late implant failure. Third, because this was a retrospective study, the two groups were not strictly randomized, and differences in fracture morphology, bone quality, comorbidities, degree of osteoporosis, and fracture location (thoracic vs. lumbar) may have influenced the outcomes. Therefore, residual confounding cannot be completely excluded. Furthermore, kyphotic angle correction and sagittal alignment maintenance were not quantified in this study because of variability in fracture morphology and postoperative imaging conditions. Nevertheless, these parameters are important radiographic indicators of spinal stability and will be included in future analyses. Fourth, the surgical procedures were performed by different surgeons, which may have introduced variability to the technique. Fifth, although all patients underwent posterior fixation alone, selection bias regarding the decision to apply TSD bilaterally or partially cannot be completely excluded. Finally, potential drawbacks of TSD fixation, including the risk of adjacent endplate injury, neurovascular injury, and technical difficulty in trajectory control, should be recognized. Careful preoperative planning and navigation assistance are essential to minimize these risks.

## 5. Conclusions

Posterior fixation using TSD demonstrated favorable outcomes in terms of blood loss, maintenance of reduction, bone union, and avoidance of reoperation in patients with DISH-related spinal fractures with marked anterior distraction. Importantly, even when an anterior gap remains postoperatively, the strong fixation achieved by TSD appears to minimize the risk of implant failure and facilitates bone union. Although further prospective, multicenter studies with larger patient cohorts are warranted, our findings indicate that this technique may be a valuable option for the surgical management of these highly unstable fractures.

## Figures and Tables

**Table 1:** Patient demographics.

	TSD Group (N = 11)	Control Group (N = 10)	*p* Value
Sex	Male 2, Female 9	Male 2, Female 8	0.916
Age (year)	81.8 ± 7.1	81.9 ± 8.6	0.981
Follow-up time (month)	15.4 ± 6.6	19.4 ± 8.4	0.236
Fracture region	Th11: 2, Th12: 1, L1: 6, L2: 1, L3: 1	Th10: 1, Th11: 1, Th12: 4, L1: 1, L2: 1, L4: 2	

**Table 2:** Surgical outcomes.

	TSD Group (n = 11)	Control Group (n = 10)	*p* Value
Number of fused segments	5 ± 1	5 ± 1	0.433
Operative time (min)	164 ± 57	168 ± 60	0.900
Blood loss (mL)	306 ± 334	528 ± 659	0.334
Screw loosening (n)	3 (27%)	6 (60%)	0.198
Bone union at final follow-up (n)	11 (100%)	8 (80%)	0.214
Implant back-out (n)	0 (0%)	4 (40%)	0.035 *
Reoperation (n)	0 (0%)	3 (30%)	0.090

* *p* < 0.05.

## Data Availability

The data presented in this study are available on request from the corresponding author due to privacy and ethical restrictions. The data include patient clinical and imaging data that cannot be made publicly available.
